# Retrospective analysis of 10 cases with esophageal fistula after anterior surgery for cervical spine fracture

**DOI:** 10.1016/j.heliyon.2023.e21244

**Published:** 2023-10-23

**Authors:** Jinpeng Du, Xiangcheng Gao, Dingjun Hao, Zhengxue Quan, Liang Yan, Baorong He

**Affiliations:** a Department of Spine Surgery, Honghui Hospital, Xi'an Jiaotong University, Friendship East Road, Xi'an City, Shaanxi Province, China; b Department of Orthopedic Surgery, The First Affiliated Hospital of Chongqing Medical University, Chongqing, China

**Keywords:** Anterior surgery, Complication, Esophageal fistula, Spine fracture

## Abstract

**Objective:**

This study aims to discuss the appropriate treatment of esophageal fistula following anterior surgery for cervical spine fracture.

**Methods:**

Clinical data of patients with cervical spine fracture treated at our research center from January 2000 to December 2019 were screened. Data of patients with esophageal fistula were included, and the causes of injury, diagnosis, and treatment were retrospectively analyzed.

**Results:**

A total of 3578 patients with cervical spine fracture were screened, among whom there were 10 cases (0.28 %) of esophageal fistula. 60 % of the cases were early-onset and all were caused by intraoperative electric knife injury. The positive rate of early endoscopy was only 25 %, while routine radiography showed a positive rate of 33.3 % after three attempts. Among the six patients with early-onset esophageal fistula, three underwent sternocleidomastoid flap transfer and two underwent primary suture, all achieving successful healing. In the four cases of late-onset esophageal fistula, two patients received implant removal, debridement, incision lavage, and sternocleidomastoid muscle flap transfer three weeks later. One patient received implant removal, debridement, vacuum sealing drainage, followed by sternocleidomastoid muscle pedicle transfer muscle flap plus lavage two weeks later and achieved complete recovery. All patients gargled alternately with metronidazole and chlorhexidine gargle after surgery.

**Conclusion:**

The occurrence of esophageal fistula is associated with surgical procedures, esophageal injury, and implant compression. Esophagography and endoscopy are the primary diagnostic methods, while incision exploration after ingestion of food mixed with methylene serves as a supplementary approach. Recommended treatments include alternating metronidazole and chlorhexidine gargles, esophageal rest, repair of the fistula, muscle flap packing, lavage and drainage, nutritional support, and removal of internal fixation if necessary. Post-surgery administration of antibiotics should continue until three consecutive lavage cultures yield negative results.

## 1 Introduction

Esophageal fistulas after anterior cervical fusion have gained attention since the first report by Balmaseda et al., in 1985 [1]. The reported incidence of esophageal fistula after anterior cervical surgery ranges from 0.04 % to 0.25 % [2,3]. With advancements in anterior cervical technology, the incidence of esophageal fistula has decreased [4]. However, this rare complication can lead to various complications including incision-site infection, mediastinal infection, purulent meningitis, and sepsis [5]. In severe cases, it can lead to the death of the patient [6]. It not only violates the concept of accelerated recovery but also consumes a lot of medical resources. Hence, understanding and effectively treating esophageal fistula is crucial. Previous studies on esophageal fistulas after anterior cervical fractures are limited, and most involve singular case reports or small case numbers [7]. Therefore, we retrospectively analyzed the detailed data of esophageal fistula treatment in 10 patients with anterior cervical fracture in our hospital over the past 20 years, summarizing the experience in injury causes, diagnostic methods, and treatments of esophageal fistula after anterior cervical fracture.

## 2 Materials and methods

Inclusion criteria: (1) lower cervical spine fracture; (2) complete hospitalization and follow-up data; (3) anterior cervical surgery (anterior fixation with plate) or combined anterior and posterior surgery; (4) patients with postoperative esophageal fistula (Pharyngalgia, fever, wound redness, and swelling appear after anterior cervical surgery, and food debris or overflowed liquids are found in the wounds after feeding in some patients). Exclusion criteria: (1) history of esophageal surgery; (2) esophageal dysplasia; (3) simple posterior surgery (cervical pedicle screw/lateral mass screw fixation); (4) patients without symptoms of esophageal injury. The study was conducted in accordance with the Declaration of Helsinki and approved by the Ethics Committee of Xi'an Honghui Hospital (202203011). All patients provided written informed consent.

Based on the inclusion and exclusion criteria, clinical data of patients with cervical spine fracture treated at our research center from January 2000 to December 2019 were collected, including causes of injury, diagnosis, treatments, and other information. The 10 patients with esophageal fistula were divided into acute (<1 week), subacute (1 week-4 weeks), and delayed (more than 4 weeks) categories based on the time from surgery to esophageal fistula. Acute and subacute esophageal fistulas were referred to as early esophageal fistulas.

## 3 Results

A total of 10 patients with esophageal fistula after anterior cervical surgery, who were admitted to our hospital from January 2000 to December 2019, were included in this study. The general information is presented in Table 1. As shown in Table 2, each case was assigned a serial number. There were 4 acute cases (P1, P2, P3, and P4), 2 subacute cases (P5 and P6), and 4 cases of delayed onset (P7, P8, P9, and P10).

**Table 1:** General information of 10 patients with esophageal fistula.

Data	Value
Age (years)	47.6 ± 7.9
Gender	
Male	9
Female	1
BMI (kg/m^2^)	25.1 ± 2.9
Fracture site	
C4	1
C5	2
C6	0
C7	4
Dislocation	3
Cause of injury	
Fall injury	5
Car accident	5
AISA grading	
A	1
B	3
C	4
D	0
E	2
Complicated with ankylosing spondylitis	7
Surgery approach	
Anterior approach	5
Anterior and posterior operation	3
Corpectomy	2

**Table 2:** Clinical data of 10 patients with esophageal fistula after an anterior approach for cervical fracture.

Categories	Serial number	Diagnosis time	Fracture site	Cause of injury	AISA grading	Surgery approach	Symptom	Diagnostic method	Site of esophageal injury	Treatment method	Complicated with ankylosing spondylitis	Bacterial culture (drug sensitivity)
Acute (<1 week)	P1	2 days after surgery	C4 burst fracture	Fall injury	A	Anterior approach	Subcutaneous emphysema	Esophagoscopy negative but laryngoscope positive	C4 level ，Electrocoagulation 1 cm	Primary suture and lavage, enteral nutrition	No	Negative
	P2	4 days after surgery	C5 fracture，C4/5 dislocation	Car accident	B	Anterior approach	Drainage tube milk	Esophagoscopy negative but laryngoscope positive	C5 level,1.5 cm	Primary suture lavage, naso-gastric tube, enteral nutrition	Yes	*Pseudomonas aeruginosa* (Tobramycin and ciprofloxacin)
	P3	4 days after surgery	C5	Car accident	C	Anterior approach	Excessive drainage, neck swelling, tenderness	Mixed food methylene blue swallowing was positive, laryngoscope was positive		Irrigation, lavage, transfer of sternocleidomastoid muscle	Yes	Bacteroides (imipenem and ceftriaxone)
	P4	7 days after surgery	C6/C7 dislocation	Car accident	C	Anterior approach	Dysphagia, swollen neck, soy sauce sample liquid 20 ml was repeatedly extracted	Methylene blue exploration was positive	Inferior margin of C7	Transfer of sternocleidomastoid muscle, lavage saline, naso-gastric tube	Yes	*Staphylococcus aureus* (coagulase positive) (vancomycin, teicoplanin)
Subacute (1 week-4 weeks)	P5	8 days after surgery	C7	Car accident	E	Anterior approach	Neck swelling, incision red, body temperature 38.7 °C	Esophagography was positive and esophagoscopy was negative	C6 0.5 cm	Saline, hydrogen peroxide, type III antibiotic flushing, lavage, enteral nutrition	Yes	α- streptococcus hemolyticus (cefazolin and ciprofloxacin)
	P6	10 days after surgery	C6/C7 dislocation	Fall injury	E	Anterior and posterior operation	Swollen neck, punctured food residue, smelly	Positive esophagography		Irrigation, sternocleidomastoid muscle flap transfer, lavage, naso-gastric tube nutrition	Yes	β- streptococcus hemolyticus (cefthiamidine and moxifloxacin)
Delayed (more than 4 weeks)	P7	5 months after surgery	C7 burst fracture	Car accident	C	Anterior and posterior operation	Fever, dysphagia	Esophagoscopy and radiography were positive, and CT suggested a mediastinal abscess	C6 level	Take fixed lavage, transfer of sternocleidomastoid muscle, lavage saline, type III antibiotics	Yes	Candida albicans (ketoconazole, fluconazole)
	P8	6 years after surgery	C5/C6 dislocation	Fall injury	B	C5/C6 corpectomy	Fever frequently. The neurological symptoms worsened	X-ray showed the internal fixation was loose, and the titanium cage shifted	C6 level	Repeated irrigation, sternocleidomastoid muscle flap transfer, lavage, naso-gastric tube	No	Aspergillus (fluconazole, amphotericin B)
	P9	7 months after surgery	C7 burst fracture	Fall injury	C	C7 corpectomy	Fever, esophagoscopy was negative	CT showed mediastinal abscess and cervical internal fixation displacement. Esophagoscopy was positive	C7 level	Gastrostomy	No	Uncultured
	P10	9 months after surgery	C7	Fall injury	B	Anterior and posterior operation	Fever frequently	esophagoscopy and radiography were positive, and CT suggested a mediastinal abscess		Take down of the anterior internal fixation. lavage, sternocleidomastoid muscle flap transfer, lavage, enteral nutrition	Yes	Candida albicans (ketoconazole, itraconazole)

## 4 Typical cases

**Fig. 1:**
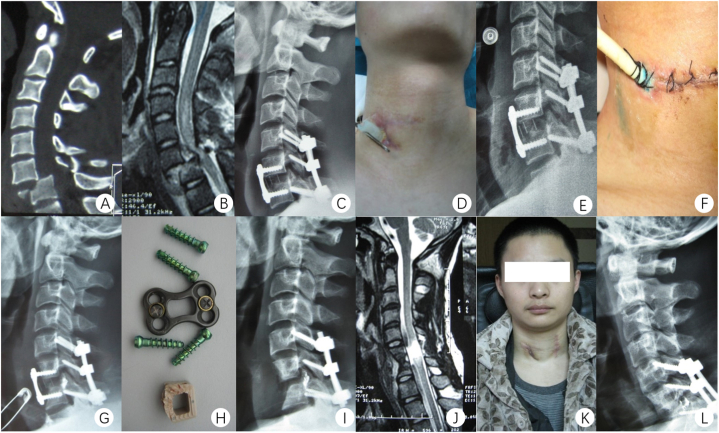
A 22-year-old male presented with a cervical fracture and dislocation (C6/7) leading to quadriplegia, classified as ASIA D. Preoperative CT (A). Preoperative MRI (B). Postoperative DR (C). Photograph of the neck incision 2 months post-surgery (D). DR at 2 months post-surgery (E). Image of incision after oral methylene blue test (F). DR at 5 months after debridement (G). A picture of the removed internal fixation (H). DR after removal of internal fixation (I). MRI 1 week after the esophageal repair (J). General picture of the patient (L). DR 5 months after the esophageal repair (K). (For interpretation of the references to colour in this figure legend, the reader is referred to the Web version of this article.)

As shown in Fig. 1, A 22-year-old male presented with neck pain following a car accident, along with impaired limb movement for a duration of 4 h. The diagnosis revealed a cervical fracture and dislocation (C6/7) leading to quadriplegia, classified as ASIA D (Fig. 1A and B). The treatment involved lateral mass screw fixation and anterior cervical discectomy and fusion (Fig. 1C). Two months post-surgery, the patient exhibited pain, redness, and swelling at the neck incision site. Incision drainage was performed, revealing a light green liquid (Fig. 1D). A digital radiography (DR) showed soft tissue thickening in front of the plate and a gas image (Fig. 1E). The diagnosis of a delayed esophageal fistula was confirmed through a positive oral methylene blue test (Fig. 1F). The patient underwent fasting, gastric tube placement, debridement, and drainage. Anti-inflammatory treatment was administered for over a month, followed by gastrostomy and jejunal nutrition tube implantation. Despite three months of conservative treatment, the sinus tract remained open. A subsequent DR at 5 months after debridement revealed bone resorption at the anterior margin of the C7 vertebra (Fig. 1G). To address this, the anterior cervical plate, screw, and cage were removed, and esophageal repair was performed (Fig. 1H and I). One week after the esophageal repair, the wound healed, and the patient resumed normal eating habits (Fig. 1J and L). Five months after the esophageal repair, radiographs showed spontaneous fusion at the C6 and C7 spaces (Fig. 1K).

**Fig. 2:**
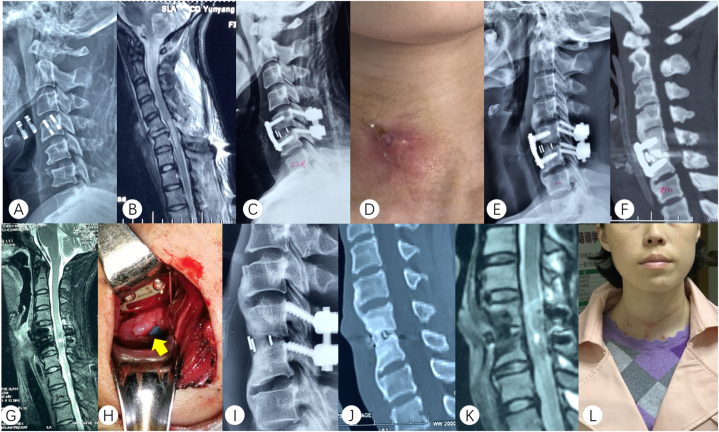
A 32-year-old female presented with a cervical fracture and cervical spinal cord injury. Preoperative DR (A) Preoperative MRI (B). DR after internal fixation treatment (C). Photograph of the neck incision 8 months post-surgery (D). DR at 8 months post-surgery (E). CT at 8 months post-surgery (F). MRI at 8 months post-surgery (G). Intraoperative photos of the second operation (H). DR at 4 months after the second operation (I). CT at 4 months after the second operation (I). MRI at 4 months after the second operation (I). General picture of the patient at 4 months after the second operation (L).

In Fig. 2, a 32-year-old female presented with a cervical fracture and cervical spinal cord injury (Fig. 2A and B). The treatment involved internal fixation using plates and screws through a combined anterior and posterior approach (Fig. 2C). At 6 months post-operation, the incision ruptured, and at 8 months post-operation, the patient experienced food residue flowing out during meals (Fig. 2D). DR and computed tomography (CT) scans at the 8-month mark showed bone resorption in front of the C5-6, fusion of the intervertebral space, and no spinal cord compression (Fig. 2E–G). To address this, the patient underwent anterior cervical plate and screw removal, as well as esophageal repair. During the operation, a tear measuring 1.1 cm was found on the posterior esophageal wall at the level of the C5 vertebral body (Fig. 2H). Imaging at 4 months post-surgery indicated sinus tract closure and bony fusion (Fig. 2I–K). The wound healed well (Fig. 2L).

## 5 Cause of injury

The cause of injury in this group was as follows: the acute fistulas (P2 and P4) occurred on the 2nd and 4th day after surgery, respectively, and were located at the levels of C4 and C5. The incision length was 1 cm and 1.5 cm, respectively, and the edges were clean, suggesting intraoperative electric knife injury. The other 4 cases of delayed esophageal fistula were mainly caused by chronic esophageal erosion due to factors such as improper placement of internal fixation, excessive bone graft, high notch, or rough edges of the cervical plate. Among these, there was a patient with C6/7 dislocation (P6) who developed esophageal fistula 10 days after surgery. A steel plate was placed to stop the esophagus, resulting in esophageal injury.

## 6 Diagnosis method

Esophagography and endoscopy were the primary diagnostic methods for esophageal fistula in this study. In addition, incision exploration after ingestion of food mixed with submethylene was used as a supplementary diagnostic method. Two patients (P1 and P2) had negative esophagoscopy results but positive laryngoscopy results. Two patients (P5 and P6) had negative routine radiography results, so oral contrast medium was administered in different positions to obtain a correct diagnosis. One patient (P7) had negative esophagoscopy, X-ray, and CT results, but positive esophagoscopy and radiography results, with CT showing a mediastinal abscess after 8 months. One patient (P4) had negative esophagoscopy and radiography results but underwent methylene blue swallow exploration. One patient (P9) had positive esophagoscopy and radiography results, with CT showing a mediastinal abscess eight months after surgery. One patient (P3) had negative esophagoscopy and CT results, negative routine swallowing, positive methylene blue swallow, and positive laryngoscopy. One patient (P9) had positive esophagoscopy after 9 months. Finally, one patient (P10) underwent removal of internal fixation, debridement, and vacuum sealing drainage (VSD), with subsequent closure of the neck swelling on the 4th day.

## 7 Treatment method

The treatment methods varied depending on the timing of esophageal fistula onset. Among the 6 patients with early-onset esophageal fistula, 3 (P4, P4, and P6) underwent sternocleidomastoid muscle flap transfer. Two patients (P1 and P2) underwent esophageal suturing in the first stage and achieved good healing. In one case (P5), the internal implant was removed, and the fistula was left open. The patient was cured after conservative treatment for 4 months. Among the 4 cases of delayed esophageal fistula, 2 (P7 and P9) underwent internal fixation removal and debridement. Three weeks after incision lavage, the sternocleidomastoid muscle flap was transferred, resulting in a cure. One case (P10) underwent internal fixation removal, debridement, VSD, and sternocleidomastoid muscle pedicled transfer muscle flap plus lavage, achieving a cure after two weeks. One case (P9) with chronic esophageal fistula received treatment in another hospital for an extended period. Gastrostomy was performed to prepare for esophagogastrostomy, but the procedure could not be completed due to economic reasons. Nevertheless, the patient survived after 5 years of follow-up. Except for P9 with gastrostomy, all patients were treated with incision lavage. Metronidazole and chlorhexidine gargles were alternated after surgery, and swallowing saliva was prohibited.

Two types of third-generation cephalosporin antibiotics were used in patients with negative bacterial cultures. The antibiotics were chosen to cover the widest possible antibacterial spectrum and were administered prophylactically for 2 weeks. For patients with positive bacterial cultures, sensitive antibiotics were selected based on drug sensitivities and administered for 2–3 weeks. Metronidazole injection and chlorhexidine gargle were alternated every 24 h, and enteral nutrition was provided via a naso-gastric tube. Three patients (P1, P2, and P5) received enteral nutrition along with intravenous nutrition due to reduced enteral nutrition solution intake caused by diarrhea. These patients were also instructed to avoid swallowing saliva and swallowing movements. The electrolyte balance was adjusted to maintain normal high levels of hemoglobin and albumin. For patients treated with antibiotics for more than 2 weeks, viable intestinal bacteria were used to regulate intestinal flora. The flushing tube was closely monitored and removed when three consecutive bacterial cultures were negative.

## 8 Discussion

Gaudianez et al. [8] reported a group of 44 patients with esophageal fistula after anterior cervical surgery, 34 of whom (77.3 %) had cervical spine fractures. In a systematic literature review by Halani et al. [7] in 2016, of the 153 patients with esophageal fistula undergoing anterior cervical surgery, 77 patients (50.3 %) had cervical fractures. These findings suggest that esophageal fistula mainly occurs in patients with cervical fractures after anterior cervical surgery, as cervical fractures can cause varying degrees of esophageal injury, and esophageal wall ischemia and traumatic reaction may be the underlying causes of esophageal fistula. Mechanical compression can easily lead to ischemia and necrosis [9]. In our study, 10 out of 3578 cervical spine injuries resulted in esophageal injuries, with an incidence rate of 0.28 %.

The most common sites for esophageal fistula are C5 and C6. The reasons for this include: (1) the esophagus lacks serosa, its posterior wall is thin, and the blood supply to the muscle layer is poor; (2) the anterior cervical plate produces higher intra-esophageal pressure at the C5-7 level compared to other cervical segments [10]; (3) the sidewall of the piriform fossa at the level of C6 is thinner, and the larger amplitude of the esophageal peristaltic wave from C5-7 is closely related to the occurrence of esophageal fistula [11,12]; and (4) as age advances, cervical degeneration, especially in the lower cervical region, can easily lead to the formation of osteophytes. When the cervical vertebra is in a state of hyperextension, it can cause compression on the anterior esophagus. All 10 patients in our study had high-energy injuries, and 90 % (9/10) had associated neurological dysfunction. Additionally, 60 % (6/10) of the patients with ankylosing spondylitis (P2, P3, P4, P5, P6, and P7) had complications related to ankylosing spondylitis. Due to the poor strain ability of the cervical vertebrae during trauma, the esophageal strain is decreased, and the pressure on the esophagus is increased. Furthermore, the osteosclerosis associated with ankylosing spondylitis can lead to the formation of sharp or broken ends after trauma, increasing the risk of esophageal compression and direct injury.

## 9 Cause analysis of injury

In our study, early esophageal fistulas (including acute and subacute) accounted for 60 % (6/10), which is consistent with the literature reports [8]. Different types of esophageal fistulas have different pathogeneses. In the early stages after surgery, sharp injuries to the esophageal wall can occur due to the operator's unfamiliarity with the local anatomy and lack of surgical skill. Prolonged pulling of the esophagus, improper strength, direction, and position of the retractor can result in local esophageal ischemia and necrosis, leading to perforation and esophageal fistula [13]. For early acute cases, the edges of the fistula are usually clean. Intraoperative electric knife injuries are generally considered as the cause.

In cases of delayed esophageal fistula, the duration is longer and the causes are varied, but they are mainly caused by chronic erosion of the esophagus by internal fixation, such as improper placement of the internal fixation, large bone grafts, a high notch of the cervical plate, and rough edges. These factors can lead to friction between the steel plate and the esophagus during swallowing, resulting in ischemic necrosis of the esophageal wall and the formation of a fistula. In addition, excessive pursuit of small incisions during surgery, unclear exposure, and accidental injuries during the operation are other contributing factors. One patient with C6/7 dislocation (P6) developed an esophageal fistula 10 days after surgery, which was believed to be due to the placement of a steel plate piercing the esophagus. In the cervicothoracic junction area, existing implant defects and poor stability can lead to loosening of the internal fixation after surgery, resulting in esophageal fistula under esophageal pressure [14]. This factor is especially prominent in cases of chronic esophageal injury. Furthermore, most patients with ankylosing spondylitis also have osteoporosis, and implant transposition and internal fixation failure are common. Fifty percent of delayed esophageal fistulas in our study were due to ankylosing spondylitis (P7, P9).

The causes of delayed esophageal fistula have not been extensively studied. Generally, factors that are not conducive to wound healing, such as infection, malnutrition, systemic inflammation, wound effusion, and endophytes, can increase the risk of esophageal fistula. Previous surgery, a history of neck radiotherapy, and long-term smoking history are also risk factors for poor healing and esophageal fistula [15]. Once this type of delayed esophageal fistula occurs, the chance of spontaneous healing is very low, unless the fistula is very small. Bacteria can easily induce the formation of a biofilm, and dysphagia can lead to reduced food intake, which in turn affects the overall nutritional status of the body. Currently, there have been advancements in material science and engineering, leading to the emergence of so-called “zero notches” anterior cervical internal fixation systems in recent years. However, there are no major reports on whether such internal fixation systems can reduce the incidence of delayed esophageal fistula.

## 10 Diagnosis method

Accurate diagnosis of esophageal fistula is crucial for effective treatment. Early diagnosis and prompt management of this complication can result in favorable clinical outcomes. However, many clinical symptoms lack specificity, making the diagnosis of esophageal fistula challenging. The main methods for diagnosing esophageal fistula in our study were esophagography, esophagoscopy, and laryngoscopy. While X-ray films can show signs such as subcutaneous emphysema, widening of the anterior marginal space of the vertebral body, and displacement and shedding of implants, the rate of missed diagnosis ranges from 10 % to 46 % [16]. CT and MRI can help determine the extent of abscess in the prevertebral space, bone destruction, location, and displacement of the internal fixation, and the positive diagnosis rate of subcutaneous emphysema is higher. Gas outside the leak, as observed through esophageal angiography, is considered the most definitive atypical clinical manifestation of esophageal leakage. When esophageal fistula is associated with vertebral infection, MRI is considered the gold standard for diagnosis, with a sensitivity of 96 % and a specificity of 93 % [16]. However, our study suggests that the diagnostic rate of MRI in the acute and subacute stages is not high.

Esophageal angiography can clearly show the location of the leak and the extent of possible spread of the exposed material. However, there is a possibility of missed diagnosis for small fistulas, with a missed diagnosis rate as high as 25 % [17]. To reduce the missed diagnosis rate of esophagography, we allowed patients to take oral contrast medium in different positions and used 200 ml iohexol to administer a large amount of contrast medium each time to improve the detection rate.

Anatomically, the esophagus at the level of the C4-6 vertebrae corresponds to the laryngopharynx. Therefore, when esophageal injury occurs above the C6 level, it is essentially a pharyngeal injury. Generally, C2 corresponds to the oropharynx, C3-6 corresponds to the laryngopharynx, and C7 and below correspond to the esophagus. Due to the entry of the esophagoscope from the mouth, it quickly passes through the laryngopharynx and cannot stay in the oropharynx and laryngopharynx. The presence of vertical folds in the esophageal mucosa requires gas injection into the esophagus to facilitate examination when using esophagoscopy to confirm the presence of esophageal injury [16,18]. However, effective inflation is not possible in these areas, limiting the value of esophagoscopy in diagnosing oropharyngeal and laryngopharyngeal injuries. Therefore, we recommend using an esophagoscope below the level of the C6/7 discs and a laryngoscope above the level of the C6/7 intervertebral discs. The laryngoscope is thin and can enter through the nasal cavity, reaching the oropharynx and laryngopharynx after passing through the nasopharynx. Although the presence of piriform recesses on both sides of the laryngopharynx can also affect the diagnosis of laryngopharyngeal injuries to some extent [17], the detection rate of esophageal fistula above the C6/7 intervertebral disc level is higher with laryngoscopy compared to esophagoscopy.

Currently, the main imaging methods for diagnosing esophageal fistula rely on positive findings from endoscopy (including esophagoscopy and laryngoscopy), esophagography, and other imaging techniques. However, the overall sensitivity of imaging examinations is only 72 %, while the positive rate of endoscopy is only 64 % [19]. Imaging examinations are generally not highly sensitive, so clinicians should be vigilant during the early stages of the disease. In addition, when performing esophagography or methylene blue swallow tests in the clinic, we use postural changes and simulate eating. If routine examinations are negative, we mix the contrast medium in 200 ml normal saline and instruct the patient to swallow a large amount to improve the detection rate. In addition, oral methylene blue and exploration through an incision are suitable for patients with subacute or late-stage disease who have negative findings on esophagogastroduodenoscopy and esophageal radiography. The timing of exploration depends on the healing status of the incision and the exclusion of other possible differential diagnoses. If early healing of the incision is poor, oral methylene blue can stain the incision or drainage tube, and repair should be performed early. In contrast, when the early postoperative incision healing is good, subacute or late manifestations of esophageal fistula, such as unexplained fever, are present, and imaging studies indicate the presence of prevertebral air and fluid, exploration and repair should be considered after excluding other possible diagnoses.

The presence of food residue in the drainage fluid after eating is the most direct evidence of esophageal fistula. Clinicians should be highly vigilant for the occurrence of esophageal fistula in the following clinical conditions [20]: (1) anterior cervical surgery in patients with cervical fractures with or without spinal cord injury; (2) early postoperative fever, elevated white blood cell count, erythrocyte sedimentation rate, and C-reactive protein levels; long-term recurrent fever with no obvious cause in the later stages; unexplained persistent tachycardia, etc.; and (3) imaging examination revealing the presence of gas or fluid in the prevertebral space or mediastinum.

## 11 Treatment

The treatment of esophageal fistula includes conservative treatment, fistula repair, and tissue reconstruction. The treatment plan depends on the size of the fistula, the time of discovery, and the presence of complications. Regardless of the chosen treatment plan, esophageal rest, wound drainage, a nasal feeding diet or enteral nutrition, parenteral nutrition, and the use of antibiotics are the basic treatments for every patient. In our study, in addition to these common treatments, we used metronidazole and chlorhexidine gargle alternately.

For esophageal fistulas discovered during surgery, primary fistula suturing should be performed, as this greatly shortens the treatment period [21]. However, for patients with large esophageal fistulas, debridement and suturing are not recommended as they may lead to secondary esophageal strictures. In such cases, muscle flap transplantation, most commonly using ipsilateral sternocleidomastoid muscle flaps, is often employed. The operative details for repairing cervical esophageal fistulas using the sternocleidomastoid muscle flap are described. After carefully separating the lower third of the sternocleidomastoid muscle, the muscle was incised from the distal clavicle, with the muscle length sufficient to cover the esophageal fistula and suture it in place. The muscle fascia is separated from the distal end and used as a reinforcing layer for closing the wound, with the help of rectus muscle and prevertebral fascia. A drainage tube is placed in the surgical area. The sternocleidomastoid muscle, with its well-vascularized, large size, and close proximity to important structures, is a common choice for head and neck plastic surgery. This technique involves cutting the muscle near the tendon and moving it to the recipient site without tension, allowing the muscle tissue to be directly sutured to the esophageal wound, and the muscle fascia can serve as a second line of reinforcement. Iatrogenic esophageal fistulas that are discovered during surgery can be repaired using this method. However, in the case of delayed esophageal fistulas, it is important to know the location of the fistula before surgery and use methylene blue to visualize the internal and external openings during the procedure. Hanwright et al. [22] compared the therapeutic effects of various types of muscle flaps and found that the repair of esophageal fistulas with free omentum muscle flaps had significantly faster healing times compared to other muscle flaps (average of 22.5 days). This may be due to the rich blood supply of the greater omentum. No cases requiring surgery were found in our study.

For early-onset esophageal fistulas (including acute and subacute), if the contamination is not severe, the local tissues around the fistula are often in good condition. Most scholars recommend early debridement and drainage without the use of a naso-gastric tube [23,24]. During the operation, we repeatedly rinsed these patients with hydrogen peroxide, metronidazole solution, and three types of amyl iodide. One case with a small fistula did not require special treatment. Two cases with large fistulas were treated with sternocleidomastoid muscle flap transfer, followed by incision lavage, resulting in good outcomes. Rueth et al. [25] also reported successful treatment of early postoperative small, mild clinical esophageal fistulas with fasting, abstinence from food, and anti-infective therapy without secondary debridement. In a patient with severe infection, bacterial culture showed Streptococcus mutans, and we removed the internal fixation during the operation. The patient was cured by repeated washing and lavage with hydrogen peroxide, metronidazole solution, and type III Amr iodine. For these patients, we treated them for 2 weeks and then observed fistula healing using swallowed methylene blue. Among the 6 patients, 4 healed, and the other 2 continued to abstain from water until three weeks after the operation, at which point all patients had healed. Therefore, we took the opportunity to gradually reintroduce water intake to these patients starting at 3 weeks after surgery. They would drink a few times over the first three days, with the irrigation tubes being observed. Three days later, they would consume pomace-free liquid for two weeks, followed by a long fiber-free semi-liquid diet for two months. After two months, they could return to a normal diet.

Delayed esophageal fistulas can have a long duration, ranging from months to years. In some patients, the incision does not heal for a long time, leading to malnutrition and mediastinal infection. It is recommended to remove the internal fixation device and perform complete debridement. This recommendation is based on the fact that pressure from the internal fixation can cause ischemia around the fistula, metallic foreign bodies can stimulate the formation of fistulas, and these patients are likely to reject metallic hardware in any case [26].

The use of antibiotics after esophageal fistula surgery is still controversial. Ardon et al. [3] reported that for esophageal fistulas discovered during surgery, repair of the fistula, postoperative fasting, and the use of nasal catheters to maintain gastrointestinal nutrition may not require the use of antibiotics. However, some authors have suggested routine use of antibiotics for approximately 7 days after surgery [27]. In our study, we cultured bacteria from the oral cavity of these patients, and the bacteria were non-definitive colonizers. The choice of antibiotics depended on the results of microbial culture and susceptibility. Before the return of bacterial culture and drug sensitivity results, antibiotics were used based on experience, usually two or three, and adjusted to sensitive antibiotics after the return of drug sensitivity results. Regardless of the availability of drug sensitivity results, antibiotics should be used in sufficient quantity and for a sufficient duration, and only stopped when the results of lavage fluid culture are negative for three consecutive times.

## 12 Prevention

The key factors for reducing complications of esophageal fistula following anterior cervical surgery include meticulous surgical technique and adherence to the following technical considerations: (i) avoiding direct esophageal injury caused by sharp instruments; (ii) preventing excessive stretching of the esophagus; (iii) avoiding compression on the esophagus by the internal fixation; and (iv) improving bone grafting and installation techniques. Surgeons should exercise particular caution to prevent esophageal fistula in patients with cervical spine injuries or cervical tuberculosis undergoing anterior cervical surgery, as they are at a higher risk of esophageal damage or inflammation [26].

In conclusion, the occurrence of esophageal fistula after anterior surgery for cervical fractures is related to the surgery itself, the injury to the esophagus, and the compression from internal implants. A comprehensive analysis and diagnosis should be based on the patient's clinical manifestations, imaging examinations, esophagography, and endoscopic examination results. We recommend using a laryngoscope for fistulas above the C6/7 intervertebral disc level and esophagoscopy for fistulas below the C6/7 intervertebral disc level. Alternating gargling with metronidazole and chlorhexidine can help reduce contamination of the fistula. With the use of esophageal fistula repair, muscle flap packing, lavage and drainage, esophageal rest, and nutritional support if necessary, as well as removal of the internal fixation, most patients can achieve satisfactory results. Postoperative antibiotics should be adjusted over time based on the results of drug sensitivity tests, following the principle of sufficient quantity and duration of treatment, and should only be stopped when the results of lavage fluid culture are negative for three consecutive times. To reduce complications of esophageal fistula following anterior cervical surgery, meticulous surgical technique and adherence to technical considerations are essential. Surgeons should exercise caution in patients with cervical spine injuries or cervical tuberculosis undergoing anterior cervical surgery to prevent esophageal fistula.

## Data availability statement

Data will be made available on request.

## Funding

This study was funded by the Project from the Science and Technology Commission of Xi’ an (21YXYG0024, 23YXYJ0083) and Science and Technology Association of Shaanxi (2021PSLK32).

## Additional information

No additional information is available for this paper.

## CRediT authorship contribution statement

**Jinpeng Du:** Funding acquisition, Data curation, Conceptualization. **Xiangcheng Gao:** Writing – original draft, Formal analysis, Data curation, Conceptualization. **Dingjun Hao:** Software, Resources, Methodology, Investigation. **Zhengxue Quan:** Software, Resources, Methodology. **Liang Yan:** Writing – review & editing, Visualization, Validation. **Baorong He:** Writing – review & editing, Investigation, Funding acquisition, Conceptualization.

## Declaration of competing interest

The authors declare that they have no known competing financial interests or personal relationships that could have appeared to influence the work reported in this paper.
